# AI and Big Data in Oncology: A Physician‐Centered Perspective on Emerging Clinical and Research Applications

**DOI:** 10.1002/cai2.70047

**Published:** 2026-01-29

**Authors:** Binliang Liu, Qingyao Shang, Jun Li, Shuna Yao, Meishuo Ouyang, Yu Wang, Sheng Luo, Quchang Ouyang

**Affiliations:** ^1^ Department of Breast Cancer Medical Oncology, Hunan Cancer Hospital/the Affiliated Cancer Hospital of Xiangya School of Medicine Central South University Changsha Hunan China; ^2^ Department of Breast Surgical Oncology, National Cancer Center/National Clinical Research Center for Cancer/Cancer Hospital Chinese Academy of Medical Sciences and Peking Union Medical College Beijing China; ^3^ Department of Applied and Computational Mathematics and Statistics, College of Science University of Notre Dame Notre Dame USA; ^4^ Department of Internal Medicine Affiliated Cancer Hospital of Zhengzhou University & Henan Cancer Hospital Zhengzhou Henan China; ^5^ Department of Surgery Duke University School of Medicine, Duke University Durham North Carolina USA; ^6^ Department of Mathematics Louisiana State University Baton Rouge Louisiana USA; ^7^ Department of Biostatistics & Bioinformatics Duke University Durham North Carolina USA

**Keywords:** artificial intelligence, big data, cancer, challenges and solutions, clinical applications, research design

## Abstract

The convergence of artificial intelligence (AI) and big data is reshaping contemporary oncology by enabling the integration of multimodal information across imaging, pathology, genomics, and clinical records. From a physician‐centered perspective, these technologies can potentially be used to improve diagnostic precision, support individualized treatment planning, enhance longitudinal patient management, and accelerate both clinical and translational research. In this review, we synthesize the core AI methodologies most relevant to oncology—machine learning, deep learning, and large language models—and examine how they interact with established and emerging oncology data platforms. We further highlight practical use cases in clinical workflows and research pipelines, emphasizing opportunities for advancing precision cancer care while also addressing challenges associated with data heterogeneity, model generalizability, privacy protection, and real‐world implementation. By underscoring the synergistic value of AI and big data, this review aims to inform the development of clinically meaningful, context‐adapted strategies that promote translational innovation in both global and locally resourced healthcare environments.

## Graphical abstract

Artificial intelligence and big data platforms are transforming oncology clinical practice. This review proposes a physician‐centered framework to integrate AI tools with real‐world data, supporting more precise diagnosis, individualized treatment, and improved patient outcomes.

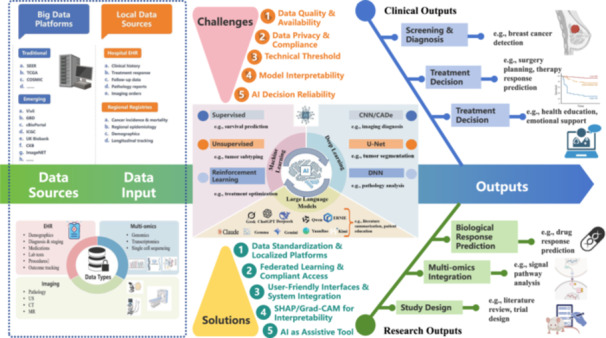

**Abbreviations**
AI: artificial intelligence
CNNs: convolutional neural networks
COSMIC: Catalog of Somatic Mutations in Cancer
DL: deep learning
DNNs: deep neural networks
EHRs: electronic health records
FL: Federated Learning
LLMs: large language models
ML: Machine learning
NCCD: National Cancer Center Database
NSCLC: non‐small cell lung cancer
TCM: traditional Chinese medicine

## 1 Current Landscape of Oncology Research

Rapid advancements in oncology have created new challenges for researchers, driven by pronounced tumor heterogeneity, exponential data growth, fragmented data systems, and fast‐paced technological evolution [1]. Cancer heterogeneity—spanning genetic, molecular, and phenotypic variations—has strengthened the demand for precision medicine. For instance, Epidermal Growth Factor Receptor mutations and Anaplastic Lymphoma Kinase rearrangements in non‐small cell lung cancer (NSCLC) necessitate tailored targeted therapies, underscoring the complexity of precision diagnostics and treatment [2]. In many countries, including China, oncology data remain scattered across institutions, with electronic health records (EHRs), imaging, pathology images, and genomic data lacking integration. Additionally, access to international databases such as the TCGA and SEER is limited for Chinese researchers due to data governance and regional policy restrictions, which further complicates data utilization [3]. Moreover, the rapid evolution of research technologies, including new experimental and statistical methods, requires continuous updates of knowledge from physicians—an ongoing challenge compounded by uneven resource distribution and high technical barriers [4].

Oncologists face dual pressures from intensive clinical workloads and increasing research expectations. According to Global Cancer Statistics 2020, China reports approximately 4.57 million new cancer cases annually, accounting for 23.7% of the global total [5], requiring physicians to make rapid decisions amid heavy clinical workloads while meeting academic and evaluation expectations. The rapid expansion and multimodal complexity of research data have outpaced the analytical capacity of humans, driving greater reliance on computational tools. In this context, artificial intelligence (AI), which has evolved over the past 70 years, is advancing at an unprecedented pace, offering new opportunities for cancer research [1, 2, 6]. Machine learning (ML) and deep learning (DL) improve diagnostic efficiency, while big data integrates heterogeneous resources to optimize treatment and research workflows, accelerating progress in oncology [1, 6].

From a physician's perspective, this review explores how AI and big data can advance oncology and support Chinese physicians in overcoming current challenges. Being a narrative review, it does not follow PRISMA guidelines; instead, it selectively integrates representative literature, illustrative cases, and physician‐centered perspectives to highlight emerging opportunities and challenges associated with AI and big data in oncology. The relevant literature and AI tools were identified through narrative, nonsystematic searches in PubMed, Google Scholar, arXiv, and CNKI, with priority given to clinically impactful and widely adopted applications, particularly those relevant to Chinese healthcare settings.

## 2 Efficient Support From AI Tools

The widespread adoption of AI technologies provides oncologists with efficient support in both research and clinical practice. By leveraging ML, DL, and large language models (LLMs), AI tools integrate diverse data sources and analyze multidimensional clinical, imaging, and molecular data to optimize workflows and accelerate research in diagnostics, drug development, and prognosis evaluation [7, 8]. From a physician's perspective, these tools not only increase diagnostic and therapeutic efficiency but also improve research accessibility and cost‐effectiveness.

### 2.1 Adoption and Impact of AI Technologies in Clinical and Research Settings

The core strength of AI technologies lies in their data processing and pattern recognition capabilities, with ML, DL, and LLMs each providing distinct and complementary support for oncologists.

#### 2.1.1 Machine Learning

ML encompasses a range of algorithms that can analyze rapidly expanding datasets and identify patterns for risk stratification and survival prediction [3, 9]. It excels in handling high‐dimensional data and uncovering nonlinear relationships, significantly increasing prediction accuracy. ML operates on principles such as supervised learning (training models with labeled data, e.g., predicting patient survival), unsupervised learning (identifying hidden patterns or associations, e.g., tumor subtype clustering), and reinforcement learning (optimizing decisions through trial and error, e.g., treatment plan optimization). Supervised learning is widely applied in survival prediction; for example, models trained with labeled data using radiomic features achieve an AUC of > 0.80 in risk stratification for NSCLC [10]. Unsupervised learning facilitates the identification of cancer subtypes and heterogeneity, such as molecular subtyping in pancreatic cancer [11]. Reinforcement learning is effective for treatment optimization; for example, radiotherapy dose allocation can be refined for NSCLC through iterative learning, thereby improving treatment precision [12].

#### 2.1.2 Deep Learning

DL employs artificial neural networks with multiple layers of simulated neurons, making it particularly adept at image and video processing. In oncology, DL is extensively used for interpreting CT and MR images and pathology slides [7, 13]. For instance, convolutional neural networks (CNNs) can automatically detect lesions in lung cancer CT images, achieving high diagnostic sensitivity and specificity, with an AUC of 0.949 [14, 15]. In pathology, CNNs can detect lymph node metastases in patients with breast cancer, with an AUC of 0.996 [16]. With respect to breast cancer screening, CNN‐driven computer‐aided detection systems outperform traditional methods because of their low sensitivity, ability to match human readers, and ability to substantially reduce physicians' workload [17]. Techniques such as lesion annotation and whole‐image labeling enable end‐to‐end training, continuously improving the accuracy of screening for breast cancer [18]. Similar DL models applied to the TCGA dataset can predict survival and recurrence with an AUC of 0.91 and a sensitivity of 98% [19], and they can analyze DNA methylation data to accurately classify disease subtypes [20]. U‐Net, for example, processes radiomic data with a screening accuracy of 93% [19]. Furthermore, during surgery, DL can analyze video feeds to identify critical anatomical structures in real time, delineate tumor boundaries, increase surgical precision and safety, and reduce errors [21, 22]. In digitized pathology slide analysis, DL demonstrates superiority in diagnosing breast, lung, and brain tumors [3, 23, 24, 25]. Through automated feature extraction and quantitative analysis, these technologies reveal complex gene‐driven mutation patterns [24] and identify potential subtypes [25], enabling precise diagnosis and providing oncologists with efficient clinical decision support [3].

#### 2.1.3 Large Language Models

Based on natural language processing, LLMs can generate text, synthesize literature, and answer questions, and they excel in literature reviews and physician–patient communication. The widespread adoption of these technologies provides physicians with comprehensive support, from data analysis to decision‐making, and has been proven to rapidly generate structured research reviews, increasing research efficiency [4, 26]. Additionally, LLMs can produce health recommendations, offering physicians and patients strategies and plans for treatment and care, and thereby assisting with routine clinical tasks [26].

### 2.2 Key AI Tools and Their Applications

To illustrate the diversity of AI applications in oncology, we summarize representative tools frequently used in clinical practice and research.

#### 2.2.1 ChatGPT

ChatGPT, which was developed by OpenAI, is a multimodal LLM with broad capability that can process text, images, and structured clinical data. It is particularly useful for literature review and hypothesis generation, enabling physicians to rapidly construct research ideas and study frameworks. Website: https://chat.openai.com.

#### 2.2.2 Grok

Grok, which was launched by xAI, is a real‐time inference model designed for rapid validation of clinical hypotheses. It can assist in evaluating antitumor drug efficacy, assessing treatment data reliability, and supporting real‐time clinical decision‐making, such as chemotherapy regimen optimization. Website: https://x.ai/grok.

#### 2.2.3 DeepSeek

DeepSeek, an open‐source LLM developed in China, offers low training costs and strong adaptability to Chinese clinical data. It is particularly suitable for processing Chinese EHRs and tumor registry data, and its strong performance in Chinese‐language generation supports oncology research in China.

#### 2.2.4 Claude

Claude, which was developed by Anthropic, is an inference‐optimized LLM with strong interpretability that can analyze complex treatment plans, generate scientific text, and increase academic writing efficiency. It is well‐suited for drafting clinical trial designs in oncology. Website: https://www.anthropic.com/claude.

#### 2.2.5 Gemma and Med‐Gemma

Gemma, which was developed by Google DeepMind, is an open‐source LLM designed for lightweight, privacy‐preserving, and scalable deployment. While Gemma itself is a general‐purpose model, it provides the architectural foundation for specialized healthcare models such as Med‐Gemma. Med‐Gemma, which was released in 2024, is a multimodal medical LLM optimized for the joint interpretation of clinical text and medical imaging data. It supports advanced tasks, including radiology report generation and visual question answering, demonstrating superior performance in multimodal medical reasoning. Website: https://ai.google.dev/gemma.

#### 2.2.6 Other LLMs Developed in China

These models demonstrate similar potential in applications in oncology, including literature synthesis, clinical data processing, and patient communication support, thereby improving both clinical and research efficiency. They generally excel at processing Chinese text. The following are several representative models and their applications.

##### 2.2.6.1 Qwen (Tongyi Qianwen, Alibaba Cloud)

Qwen, which is an open‐source multimodal LLM, excels at processing Chinese text, images, and data, which makes it suitable for generating literature reviews in oncology, analyzing EHRs, and optimizing clinical trial designs. Its low cost and high performance support a wide range of enterprise applications. Website: https://www.aliyun.com/product/qwen.

##### 2.2.6.2 ERNIE Bot (Wenxin Yiyan, Baidu)

ERNIE Bot, which is a Chinese‐optimized LLM, supports intelligent consultations and literature processing, providing health advice to cancer patients (e.g., addressing chemotherapy side effects) or generating cancer research reports, thereby improving diagnostic and therapeutic communication efficiency. Website: https://yiyan.baidu.com/.

##### 2.2.6.3 KIMI (Moonshot AI)

KIMI, which is an efficient Chinese LLM, focuses on long‐text processing and knowledge retrieval, which makes it suitable for rapidly accessing oncology research updates or generating structured research documents, such as reviews on targeted therapies. Website: https://kimi.moonshot.cn/.

##### 2.2.6.4 Tencent Yuanbao (Tencent)

Tencent Yuanbao, which is a multimodal LLM based on the Hunyuan model, supports literature summarization, patient education, and multimodal data analysis. These uses make it suitable for generating oncology patient guidance materials or analyzing treatment outcomes. Website: https://yuanbao.tencent.com/.

##### 2.2.6.5 Other Distinctive LLMs

###### 2.2.6.5.1 DISC‐MedLLM [27]

DISC‐MedLLM, which was designed specifically for medical dialog scenarios, is an LLM based on the Baichuan‐13B model. It is fine‐tuned with high‐quality medical knowledge graphs and real dialog datasets. It excels at single‐ and multiturn medical consultations and can be used for patient inquiries, generating clinical records, or supporting telemedicine. Website: https://www.baichuan-ai.com/home (company website).

###### 2.2.6.5.2 Zhongjing [28]

ZhongJing, which is a domain‐specific LLM designed for traditional Chinese medicine (TCM), is fine‐tuned in the TCM field to support TCM diagnosis, treatment assistance, and training. In oncology, ZhongJing can be used to analyze TCM literature, generate cancer‐related TCM treatment plans (e.g., optimizing herbal prescriptions), or support patient health education. Website: Not available (academic project).

## 3 Big Data Platforms: Physicians' Perspective on Data Resources

Big data platforms are designed to store, integrate, and analyze large‐scale medical data, covering molecular omics (genomics, transcriptomics, epigenetics, proteomics, and metabolomics), perturbed phenotypes, molecular interactions, imaging, and clinical text data [3]. For instance, EHRs document patient histories and treatment responses, multiomics data reveal tumor molecular profiles, and imaging data aid in lesion localization [3]. From a physician's perspective, these platforms increase diagnostic precision and expand research resources, while localized platforms are particularly valuable in China considering the access to many international databases is restricted. Big data platforms can be broadly categorized into traditional and emerging types, each serving distinct research and clinical applications (Table 1).

**Table 1:** Comparison of big data platforms in oncology.

Platform	Data coverage	Sample size	Cancer types covered	Access level	Population representation	Clinical linkage	Strengths	Limitations	Website
SEER [29]	U.S. cancer epidemiology	~10 M cases	All major cancers (U.S.)	Open	U.S. population	Yes	High‐quality, standardized, survival analysis	Western‐focused, differs from Chinese profiles	https://seer.cancer.gov
TCGA [30]	Multi‐omics	~11,000 samples	33 cancer types	Open	Primarily U.S.	Partial	Core resource for molecular research	Slow updates, limited sample size (11,000)	https://cancergenome.nih.gov
GEO [31]	Multi‐omics	~6 M samples	Multiple (user‐submitted)	Open	Global	No	Diverse data, frequent updates, user uploads	Low standardization, inconsistent annotations	https://www.ncbi.nlm.nih.gov/geo
COSMIC [32]	Somatic mutations	> 37,000 genomes	Various somatic mutation data	Open	Global	No	Large data volume, molecular target research	Limited clinical information	https://cancer.sanger.ac.uk/cosmic
NCCD/NCDL [33]	Chinese cancer registry	Nationwide registry	All major cancers (China)	Restricted	Chinese	Limited	Convenient access, localized research	Macro‐level, lacks multi‐omics details	https://www.ncc.org.cn
OncoKB [34]	Genomic variants, therapeutic implications	Variant‐based	Therapeutically relevant cancers	Open	Global	Yes	Precision oncology, actionable insights	Limited to annotated variants	https://www.oncokb.org
GDC [35]	Cancer genomics	TCGA, TARGET, etc.	Multiple cancer types	Open	Primarily U.S.	Partial	Standardized, supports data sharing	Primarily U.S.‐based data	https://gdc.cancer.gov
ArrayExpress [36]	Functional genomics	Millions	Broad (user‐submitted)	Open	Global	No	Diverse data, user submissions	Inconsistent annotations	https://www.ebi.ac.uk/arrayexpress
Vivli [37]	Global clinical trial data	Thousands of trials	Varies by trial	Controlled	Global	Yes	High‐quality, cloud‐based analysis	Strict compliance, high entry barrier	https://vivli.org
GBD [38]	Global disease burden	Global estimates	All major cancers	Open	Global	No	Authoritative, macro‐level research	Lacks individualized clinical details	https://www.healthdata.org/gbd
cBioPortal [39]	Cancer genomics	~200 studies	Broad (mostly TCGA‐linked)	Open	Primarily Western	Yes	Interactive visualization, mutation analysis	Western‐focused, limited global representation	https://www.cbioportal.org
ICGC [40]	Cancer genomics	~20,000 samples	50+ cancer types	Open	Global (incl. China)	Partial	Dynamic updates, includes Chinese data	Limited clinical annotations	https://dcc.icgc.org
UK Biobank [41]	Genomics, phenotypes	500,000 individuals	Various (population study)	Controlled	UK	Yes	Open‐access, cloud analysis (UKB‐RAP)	Western‐focused	https://www.ukbiobank.ac.uk
CKB [42]	Chinese health data	500,000 individuals	Various (Chinese population)	Restricted	Chinese	Yes	Rich localized data	Limited multi‐omics depth	https://ckbiobank.pku.edu.cn
ImageNet [43]	Annotated images	14 M images	Not cancer‐specific	Open	Non‐medical	No	Large volume, supports AI pre‐training	Non‐medical, requires transfer learning	https://www.image-net.org
CIViC [44]	Clinical interpretations of cancer variants	Thousands of variants	Cancer variants (clinical)	Open	Global	Yes	Community‐driven, actionable insights	Limited scale, ongoing curation	https://civicdb.org
CCLE [45]	Cancer cell line multi‐omics	~1000 cell lines	Many cancer types (preclinical)	Open	Cell lines	No	Drug response, molecular profiling	Cell line‐based, lacks patient context	https://portals.broadinstitute.org/ccle
EGA [46]	Genomic and phenotypic data	Hundreds of studies	Various (Europe‐focused)	Controlled	European	Partial	Secure access, supports cancer research	Restricted access, complex application	https://ega-archive.org
DepMap [47]	Cancer dependency genes, drug targets	> 1000 cell lines	Cancer dependency/target discovery	Open	Cell lines	No	Functional genomics, drug screening	Cell line‐focused, limited clinical data	https://depmap.org
PCAWG [30]	Pan‐cancer whole genomes	~2,800 genomes	Pan‐cancer	Open	Global	Partial	Comprehensive genomic insights	Complex data, limited clinical integration	https://dcc.icgc.org/pcawg

### 3.1 Traditional Big Data Platforms

Traditional platforms typically contain regional or institution‐based datasets with relatively standardized data collection and long‐term follow‐up, which makes them foundational resources for oncology research. Notable examples include the SEER, TCGA, GEO, and Catalog of Somatic Mutations in Cancer (COSMIC) databases and the National Cancer Center Database (NCCD), which provide standardized data for epidemiological and molecular studies but often lack global representativeness or detailed clinical annotations (see Table 1 for details).

### 3.2 Emerging Big Data Platforms

Emerging platforms leverage cloud computing, cross‐institutional data sharing, and interactive analytical tools to provide dynamic and scalable support, representing the future direction of data integration in oncology. Platforms such as Vivli, GBD, cBioPortal, ICGC, UK Biobank, CKB, and ImageNet offer expanded data coverage and cloud‐based analytical capabilities but may be limited by access policies, population bias, or insufficient clinical depth (see Table 1 for details).

## 4 Empowering Clinical Practice and Research With AI and Big Data

The synergistic integration of AI and big data technologies, particularly through multimodal data analysis, significantly increases the precision and efficiency of oncology practice while simultaneously advancing research. Big data platforms such as COSMIC, TCGA, and the UK Biobank, in addition to high‐quality localized institutional databases, provide vast standardized datasets, while AI algorithms extract insights via DL and ML. Together, they enable comprehensive optimization across the entire continuum—from tumor screening and diagnosis to treatment decision‐making, as well as from research design to outcome evaluation [3].

### 4.1 Empowering Clinical Practice

#### 4.1.1 Cancer Screening and Imaging Diagnosis Support

Advances in diagnostic imaging and biomedical image analysis have enabled unprecedented access to structural and metabolic information. AI integrates multimodal data, including imaging (e.g., X‐ray, ultrasound, and MRI), EHRs, and molecular profiles (e.g., gene expression and mutation status), thereby significantly improving diagnostic accuracy [1, 10, 48]. AI demonstrates a core advantage in image analysis, leveraging standardized imaging data from platforms such as CBIS‐DDSM and the UK Biobank and genomic insights from the TCGA. When DL is used, AI algorithms can rapidly detect lesions, reduce misdiagnosis rates, and achieve diagnostic performance comparable to that of experienced specialists [4, 49, 50].

AI excels at breast cancer screening, particularly in detecting microcalcifications, which are early lesion markers [48]. Studies using the Transpara AI system to analyze mammography images from the CBIS‐DDSM dataset achieved automated detection of microcalcifications and soft tissue lesions, with an AUC of 0.84, outperforming the radiologists by 61.4% [50, 51]. Shen et al. [18] further validated the utility of AI in ultrasound imaging and developed an “end‐to‐end” CNN based on the CBIS‐DDSM dataset, achieving an AUC of 0.91. Fine‐tuning further increased diagnostic accuracy and reduced the number of unnecessary biopsies. Additionally, AI combined with digital breast tomosynthesis data has been shown to improve early breast cancer detection sensitivity. Using institutional datasets, Geras et al. [52] reported an AUC of 0.87 with AI models in DBT, minimizing the number of false positives. Dynamic contrast‐enhanced MRI, a DL model developed by Ayatollahi et al. [53] that is based on the Radboud University Medical Center breast MRI dataset, analyzed the spatiotemporal patterns of contrast uptake, enabling ultrafast lesion segmentation with an AUC of 0.85.

These results underscore the dependence on standardized imaging data from CBIS‐DDSM or other high‐quality databases, highlighting the synergy between AI algorithms and high‐quality data in yielding superior models.

#### 4.1.2 Support for Tumor Pathology Diagnosis

AI applies deep neural networks (DNNs) to digitized pathology slides, demonstrating high efficiency and consistency in diagnosing cancers such as breast, lung, and brain tumors [1, 23, 24, 25]. For instance, Bejnordi et al. [23] confirmed that DNNs reduce the diagnosis time and improve the classification consistency when large pathology datasets are used. In lung cancer, AI utilizes whole‐slide images from the TCGA to accurately classify cancer types and detect specific gene‐driven mutation patterns that are undetectable by traditional pathologists [24]. Additionally, AI leverages tumor DNA methylation patterns from large sequencing datasets for ML, markedly improving the accuracy of brain tumor subtype classification over that of traditional histological methods [25].

#### 4.1.3 Precision Therapy and Personalized Treatment Decision‐Making

By integrating multimodal data from big data platforms, along with clinical and treatment response data, AI significantly optimizes breast cancer treatment decision‐making, increases clinical precision, and improves patient management efficiency.

In surgical planning, AI uses DL to analyze MR and CT images and automatically segments tumor boundaries to assist in preoperative planning and intraoperative navigation for patients with breast cancer. For example, Zhang et al. achieved tumor segmentation with an AUC of > 0.90 using MRI data, reducing the reliance on manual annotations [54]. CNNs can process intraoperative videos to identify critical anatomical structures in real time during colorectal surgery or liver resection, increasing surgical precision and safety while minimizing errors [21]. In precision breast cancer subtyping, Jiang et al. [55] integrated multidimensional data—genomics, transcriptomics, proteomics, metabolomics, and radiomics—from 1226 breast cancer patients, proposed molecular subtypes for luminal breast cancer, and achieved accurate predictions using AI models. In treatment response prediction, AI integrates multiomics data (e.g., mutation burden and immune infiltration) to predict the pathological complete response to neoadjuvant chemotherapy. For example, Sammut et al. [56] used data from 168 patients to develop an integrated ML model that achieved an AUC of 0.87 in external validation (75 patients), outperforming traditional models. DL architectures such as U‐Net architectures precisely delineate lesion boundaries in breast MR images, reducing the reliance on manual expert annotations and improving the efficiency of radiotherapy planning [57]. Through feature selection and multiomics integration, AI has demonstrated substantial potential in breast cancer biomarker discovery and application, providing robust support for precision diagnosis and personalized treatment. A model combining the gray wolf optimization algorithm with support vector machines improved diagnostic accuracy by 27.68% on the TCGA breast cancer dataset by optimizing gene expression features. Similarly, the multiomics graph convolutional network, which integrates TCGA gene expression, DNA methylation, and miRNA data, achieved an F1 score of 0.89 for breast cancer subtype classification, supporting targeted therapies such as HER2‐directed drugs [58]. The CURATE. AI platform used in chemotherapy for advanced solid tumors can dynamically adjust capecitabine doses on the basis of patient biomarkers (e.g., PSA levels in prostate cancer), improving quality of life and disease control rates and offering a promising approach for managing adverse reactions in the future [59].

AI also facilitates rapid retrieval of the latest treatment guidelines, using natural language processing to access breast cancer protocols, assisting physicians in developing standardized plans, predicting adverse drug reactions, and identifying rare drug interactions to increase treatment safety [26].

#### 4.1.4 Patient Management

LLMs enhance oncology patient management by providing online consultations, health education, and emotional support, thus significantly improving patient self‐management and reducing clinical burdens. LLMs provide personalized information on disease, treatment, and prevention for breast cancer patients, equipping them with essential knowledge before clinical consultations [60]. During treatment, LLMs facilitate effective physician–patient communication and health education. For instance, Bibault et al. [61] reported that AI chatbots provide breast cancer information comparable to that provided by physicians, alleviating clinical workloads. ML‐based health management platforms integrate multimodal data (e.g., heart rate and sleep data from wearable devices and EHRs) to create customized care plans, such as exercise recommendations or medication adherence reminders [2, 62, 63]. LLMs also generate health recommendations, offer personalized care advice, develop individualized care plans [64], and provide emotionally supportive dialogs to reduce patients' psychological stress and promote adherence [65, 66], thereby enabling effective self‐management and substantially lowering hospital management and care costs.

### 4.2 Empowering Research

In research, AI and big data provide efficient support for physicians from study design to outcome generation, with particular strengths in localized data processing:

#### 4.2.1 Study Design, Writing, and Figure Generation

LLMs such as ChatGPT rapidly acquire multidisciplinary data and efficiently retrieve the latest studies from medical literature to assist researchers in synthesizing literature, drafting trial protocols, and constructing manuscript frameworks, thereby facilitating research [4, 26, 67]. LLMs such as Grok leverage real‐time literature validation to track research trends and emerging evidence, helping ensure that studies remain current and meet publication standards. Additionally, the ability of AI to automatically generate statistical figures and mechanistic diagrams increases manuscript visual quality and supports physicians in conducting high‐quality research, overcoming knowledge barriers, and promoting innovative outputs across research institutions.

#### 4.2.2 Breakthroughs in Cross‐Modal Integration and Cross‐Cohort Aggregation

The integration of AI with big data enables breakthroughs in elucidating cancer molecular mechanisms and optimizing diagnostics by cleaning heterogeneous data and fusing multimodal datasets. AI models integrate genomic, transcriptomic, and proteomic data to elucidate key signaling pathways. Single‐cell multiomics technologies (e.g., scRNA‐seq combined with scATAC‐seq), analyzed via AI, significantly increase the accuracy of cell lineage classification in the breast cancer microenvironment [68]. Studies have shown that integrating scRNA‐seq and scATAC‐seq data with AI methods, such as graph neural networks, effectively identifies cancer‐associated fibroblast subpopulations (e.g., inflammatory and matrix cancer‐associated fibroblast), outperforming traditional methods. Cross‐cohort, multicenter data integration mitigates the noise and bias inherent in single datasets, markedly improving the identification of key driver genes and disease‐associated mutations [69].

#### 4.2.3 AI Prediction of Biological Responses

**Figure 1:**
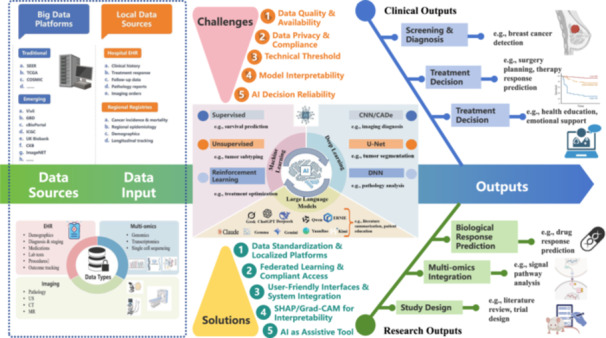
Physician‐centered closed‐loop framework for the integration of AI and big data into oncology practice. In this model, multimodal patient information—including structured clinical records, radiological and pathological imaging, and genomic or molecular profiling—is systematically collected and standardized through hospital information and big‐data platforms. AI models—including machine learning (ML), deep learning (DL), and large language models (LLMs)—trained on these datasets generate diagnostic support, risk stratification outputs, and therapeutic recommendations. Rather than functioning as passive end users, physicians critically evaluate AI outputs, contextualize them within clinical judgment, and adapt decision‐making accordingly. Their clinical feedback and outcome observations are then iteratively incorporated into model retraining and refinement. This bidirectional interaction forms a dynamic learning cycle, ensuring that the AI system remains clinically aligned, achieves progressively improved performance, and evolves in response to real‐world oncological needs.

AI effectively predicts the activity, toxicity, and pharmacokinetic properties of new drug compounds by analyzing multiomics data, including genomics, metabolomics, and proteomics data, offering early screening potential that significantly shortens development timelines and reduces costs [70, 71]. AlphaFold3 surpasses existing specialized tools in predicting protein–ligand, protein–nucleic acid, and antibody–antigen interactions, demonstrating increased accuracy. Additionally, AlphaFold3 can predict the structural impact of posttranslational modifications on molecular systems, offering a powerful tool for drug design, biotechnology, and fundamental biological research. This model achieves high‐precision modeling across biomolecular spaces through a unified DL framework [72]. In breast cancer drug repurposing, AI integration of multiomics data also has notable advantages: the NCI‐DREAM challenge employs multikernel learning and combines genomic, transcriptomic, and proteomic data from breast cancer cell lines to predict drug responses, outperforming single‐omics models [4, 73]. Furthermore, random forests and support vector machines achieved an AUC of 0.90 in predicting chemotherapy responses on the GDSC dataset, whereas DNNs predicted drug sensitivity on the CCLE dataset with an AUC of 0.91 [19, 74]. These findings highlight the substantial potential of AI in predicting biological responses (Figure 1).

## 5 Challenges and Solutions for AI and Big Data in Oncology

A key emerging opportunity emphasized in this review is the shift from physicians serving as passive users of AI tools to becoming active participants in model development, evaluation, and ongoing optimization. In clinical oncology, physicians possess irreplaceable contextual knowledge regarding disease progression, treatment responses, and patient‐specific factors. Integrating this expertise into AI model refinement establishes a physician‐centered feedback loop, enabling AI systems to better align with real‐world clinical decision‐making. This collaborative model has the potential to substantially improve model interpretability, clinical relevance, and patient outcomes, representing a meaningful evolution in the implementation of AI in oncology practice.

Despite their transformative potential, the adoption of AI and big data technologies in oncology is hindered by multiple challenges. Current barriers include heterogeneous data quality, stringent privacy regulations, technical limitations, limited interpretability of models, and uncertainty regarding reproducibility [3]. To mitigate these issues, several strategies have emerged, including federated learning (FL) to enable privacy‐preserving data sharing, standardized data pipelines, user‐centered interface design, and explainable AI frameworks. Collectively, these approaches can foster physician trust and facilitate the integration of AI into precision oncology.

### 5.1 Challenges

#### 5.1.1 Data Quality and Availability

Traditional databases such as the COSMIC focus on somatic mutation data across diverse cancer types but lack comprehensive clinical and multiomics information, limiting their utility in precision medicine [32]. Emerging platforms such as cBioPortal offer interactive genomic analysis but are based primarily on Western samples, potentially underrepresenting global populations, such as Asians with a high prevalence of EGFR mutations [75]. Additionally, data from different centers are often fragmented and insufficiently standardized, undermining the reliability of data integration and analysis [1]. Moreover, the overall scale of cancer data remains limited, with measurement inconsistencies and batch effects further impacting analytical accuracy [3].

#### 5.1.2 Data Privacy and Compliance

The biomedical field maintains limited data openness, with privacy regulations posing a major obstacle [3]. China's Data Security Law [76] and Personal Information Protection Law prohibit the cross‐border transfer of sensitive data without approval [77]; similarly, the U.S. Executive Order 14117 (issued in February 2024) restricts access to sensitive personal U.S. data and government‐related data for “countries of concern”, including China, increasing the difficulty of accessing databases such as SEER or the TCGA and hindering international research collaboration and publication. The unique nature of the medical field also demands caution when new technologies are introduced [78]. Despite the robust data processing and integration capabilities of AI, prediction errors persist in clinical settings [79].

#### 5.1.3 Technical Barriers

Oncologists often lack a data science background, making it challenging to navigate complex platforms and thus limiting the adoption of technology [80]. For instance, cBioPortal's genomic visualization requires programming skills [39], and Vivli's cloud‐based analysis demands familiarity with data processing workflows, restricting direct use by clinicians. Additionally, many AI tools operate as standalone systems and struggle to integrate seamlessly with EHRs or picture archiving and communication systems (PACSs). This often requires manual data transfer or uploading, increasing physicians' workload and further hindering widespread adoption [81].

#### 5.1.4 Lack of Model Interpretability

DL models often lack interpretability because of their “black box” nature, with opaque decision‐making processes that prevent physicians from understanding the basis of predictions [82]. This reduces the degree of trust among clinicians and patients and limits the translation of AI models into clinical practice.

#### 5.1.5 Model Reliability and Generalizability

Medicine is an inherently complex field compounded by issues such as outdated data. Current language models exhibit limitations in terms of contextual understanding and causal reasoning for complex medical decisions and require caution to avoid misleading recommendations [83, 84]. While AI diagnostic models perform well in specific research settings, their training data often come from single institutions or datasets, leading to poor generalizability across diverse healthcare settings and populations and resulting in unstable performance. Unvalidated AI predictions may result in errors, as models remain vulnerable to real‐world data variability, thereby reducing their robustness [4, 26].

### 5.2 Solutions to Address Challenges

#### 5.2.1 FL and Privacy Protection

FL enables the integration of multicenter data while safeguarding privacy through local training and model parameter sharing, making it well suited for oncology research [85, 86]. It incorporates techniques such as differential privacy, homomorphic encryption, and cryptonets to reduce data leakage risks and facilitate secure multicenter collaboration [87]. Unlike traditional centralized training, FL avoids data aggregation, further reducing the risk of parameter leakage and ensuring patient data security. For example, multiple hospitals can share updates to breast cancer treatment response models, increasing the diagnostic accuracy for rare tumors [63, 64, 65, 66]. Additionally, cross‐border collaboration and compliant data access (e.g., through anonymized data sharing) ensure that platforms such as Vivli and SEER meet regulatory requirements.

#### 5.2.2 Data Standardization and Localized Platforms

Standardizing data formats across platforms or research centers and addressing missing values can significantly reduce the complexity of data integration, thereby improving data quality. For instance, AI tools such as DeepSeek utilize natural language processing to automate the cleaning of heterogeneous data, enhancing the research utility of platforms such as COSMIC and cBioPortal [26]. The development of localized platforms, such as ICGC and NCCD, helps address gaps in population representation; ICGC, for example, integrates Asian population data, which makes it ideal for studying regional mutation patterns [33]. Optimizing these platforms to support diverse population data further enhances their global applicability.

#### 5.2.3 User‐Friendly Interfaces and System Integration

Designing simplified data input and analysis interfaces can lower technical barriers for users. For example, Vivli's interactive cloud platform enables nonexpert users to conduct research, whereas cBioPortal's visualization tools allow physicians to analyze genetic mutations easily [75]. DeepSeek's intuitive interface supports chat‐based interactions and document uploads, enabling primary care physicians to analyze EHRs or imaging data without programming skills. Embedding AI tools within EHR and PACS systems provides real‐time clinical decision support, such as automated lesion annotation, thereby reducing manual workload [88, 89]. Additionally, lightweight AI models leveraging edge computing can operate on local devices in primary hospitals, analyze ultrasound images in real time and address disparities in medical resources [90].

#### 5.2.4 Enhancing Model Interpretability

To improve model interpretability, tools such as SHapley Additive Explanations quantify feature importance by showing the contribution of specific risk factors to prognosis, helping physicians understand model decisions [91]. Similarly, gradient‐weighted class activation mapping generates imaging heatmaps to highlight AI‐identified lesion areas, assisting radiologists in validating predictions [92]. These interpretability tools markedly improve clinical acceptance, foster physician trust, and support broader implementation [93, 94].

#### 5.2.5 AI Decisions as Assistive Only

AI should function as an assistive tool in clinical and research decision‐making—particularly for initial screening or hypothesis generation—enhancing rather than replacing human judgment. AI predictions are best suited for early‐stage exploration or brainstorming, with an emphasis on interpretability and quality monitoring. To improve robustness, subsequent validation using cross‐cohort or independent external datasets is essential [4, 95].

## 6 Future Directions: Physicians' Expectations and Technological Outlook

The rapid advancement of AI and big data technologies presents substantial opportunities for the future of oncology. Clinically, AI increases diagnostic precision and treatment efficiency by aiding diagnosis, optimizing therapeutic decisions, and improving patient management. In research, AI and big data accelerate study design, data analysis, and outcome generation, promoting localized research development. The future of “AI + big data” depends not only on technological breakthroughs but also on balancing societal trust with medical ethics, fostering synergy between localization and internationalization, and redefining the evolving role of physicians [96, 97]. Currently, big data platforms in oncology must increase sample sizes and promote data sharing [3, 16] while increasing the digitization of laboratory tests, imaging, and pathology slides [1, 98, 99]. Multimodal AI, generative AI, and edge computing will be pivotal in advancing precision medicine.

Multimodal AI integrates imaging, genomic, and clinical data to provide comprehensive diagnostic and therapeutic support, substantially increasing clinical precision [4]. Generative AI leverages multisource data and standardized platforms to advance tumor drug repurposing, accelerate novel drug development, and optimize combination therapies through toxicity prediction models [71, 72, 73]. Edge computing enables the local deployment of AI on devices, reducing reliance on cloud systems and improving real‐time performance, such as enabling immediate lesion detection during ultrasound examinations in primary hospitals and minimizing data transfer delays—an approach particularly suitable for resource‐limited settings [100]. These technologies will markedly improve the precision and efficiency of tumor management, especially in primary care settings.

The parallel development of localization and internationalization will be essential in the future. Localized applications can utilize NCCD and CKB data, with FL ensuring privacy while analyzing Chinese population characteristics. Moreover, localized systems can harness EHRs from Chinese hospitals and the NCCD to develop models tailored to local populations, addressing the limitations of restricted international data access. Intelligent service platforms integrating EHRs, omics, and mobile health data can support patient self‐management and clinical decision‐making, improve model applicability, and provide accessible solutions for primary hospitals, thus promoting equitable medical resource distribution.

The evolving role of physicians will be central to the integration of AI into clinical oncology. Historically, physicians functioned largely as passive adopters, depending on engineers for technical implementation. Looking ahead, however, clinicians are expected to become active innovators—engaging in interdisciplinary collaborations, shaping trial design, and contributing to model development and validation. This transition represents a paradigm shift in which physicians are not only end‐users but also active cocreators of AI‐enabled precision oncology. For example, by refining model input features with clinical expertise or reannotating AI outputs, physicians can increase the accuracy and applicability of AI in clinical settings [16, 18]. Leveraging the ability of AI to continuously improve with new data, a closed‐loop “data−prediction−feedback” system can be established that incorporates real‐time clinical data (e.g., imaging and laboratory results) and physician feedback into iterative model training, creating dynamically updated diagnostic support tools [54]. Achieving this vision requires a collaborative ecosystem in which physicians, engineers, and data scientists work together: engineers develop technical frameworks, physicians provide clinical insights, and data scientists optimize algorithms to create AI systems tailored to medical needs. Cloud collaboration and localized model optimization further support this ecosystem, with tools such as edge computing and DeepSeek enabling lightweight AI deployment in primary hospitals for real‐time diagnostics [85].

In summary, the future of oncology will be shaped by multimodal and generative AI, edge computing, and localized applications that integrate national databases such as the NCCD. Physicians will be not only users but also cocreators of these systems, ensuring that technological advances align with patient needs and clinical realities.

## 7 Conclusion

This review from a physician's perspective highlights how AI and big data are reshaping oncology by accelerating research, improving diagnostic accuracy, and supporting patient‐centered care. Localized platforms such as DeepSeek and the NCCD, together with FL and edge computing, offer practical approaches to address China's unique challenges in terms of data security and resource distribution. However, realizing the full potential of these technologies requires careful and responsible implementation. Without adequate safeguards for privacy, interpretability, and data quality, premature adoption may compromise both patient safety and healthcare equity.

Looking ahead, the dual pathway of localization and internationalization will continue to strengthen China's role in global oncology. As physicians evolve from technology users to active collaborators and innovators, they will remain central to ensuring that AI is responsibly implemented to advance precision oncology and improve patient outcomes.

## Author Contributions

**Binliang Liu:** writing – review and editing, writing – original draft, funding acquisition, conceptualization, data curation. **Qingyao Shang:** conceptualization, writing – original draft, writing – review and editing, data curation. **Jun Li:** writing – original draft, writing – review and editing. **Shuna Yao:** writing – original draft, writing – review and editing. **Meishuo Ouyang:** writing – original draft, writing – review and editing, data curation. **Yu Wang:** writing – original draft, writing – review and editing. **Sheng Luo:** writing – original draft, writing – review and editing, supervision, conceptualization. **Quchang Ouyang:** writing – original draft, writing – review and editing, conceptualization, supervision.

## Consent

The authors have nothing to report.

## Conflicts of Interest

The authors declare no conflicts of interest.

## Data Availability

Data sharing is not applicable to this article as no datasets were generated or analyzed during the current study.
